# Review on the role of the human Polyomavirus JC in the development of tumors

**DOI:** 10.1186/s13027-017-0122-0

**Published:** 2017-02-03

**Authors:** Serena Delbue, Manola Comar, Pasquale Ferrante

**Affiliations:** 1 Department of Biomedical, Surgical and Dental Sciences, University of Milano, Via Pascal, 36-20133 Milan, Italy; 2 Department of Medical Sciences, University of Trieste, Trieste, Italy; 3 Institute for Maternal and Child Health-IRCCS “Burlo Garofolo”, 34137 Trieste, Italy; 4 Istituto Clinico Città Studi, Milan, Italy

**Keywords:** JC virus, Central nervous system tumors, Colon cancer

## Abstract

Almost one fifth of human cancers worldwide are associated with infectious agents, either bacteria or viruses, and this makes the possible association between infections and tumors a relevant research issue. We focused our attention on the human Polyomavirus JC (JCPyV), that is a small, naked DNA virus, belonging to the *Polyomaviridae* family. It is the recognized etiological agent of the Progressive Multifocal Leukoencephalopathy (PML), a fatal demyelinating disease, occurring in immunosuppressed individuals.

JCPyV is able to induce cell transformation in vitro when infecting non-permissive cells, that do not support viral replication and JCPyV inoculation into small animal models and non human primates drives to tumor formation. The molecular mechanisms involved in JCPyV oncogenesis have been extensively studied: the main oncogenic viral protein is the large tumor antigen (T-Ag), that is able to bind, among other cellular factors, both Retinoblastoma protein (pRb) and p53 and to dysregulate the cell cycle, but also the early proteins small tumor antigen (t-Ag) and Agnoprotein appear to cooperate in the process of cell transformation.

Consequently, it is not surprising that JCPyV genomic sequences and protein expression have been detected in Central Nervous System (CNS) tumors and colon cancer and an association between this virus and several brain and non CNS-tumors has been proposed. However, the significances of these findings are under debate because there is still insufficient evidence of a casual association between JCPyV and solid cancer development.

In this paper we summarized and critically analyzed the published literature, in order to describe the current knowledge on the possible role of JCPyV in the development of human tumors.

## Background

The Human Polyomaviruses (hPyV) are small, naked viruses with icosahedral capsid and circular, double-stranded DNA genome. They belong to the *Polyomaviridae* family and are able to infect and establish latency in the human host. The name “Polyomavirus” derives from the Greek roots poly-, which means “many”, and –oma, which means “tumors”. To date, at least thirteen human members of the *Polyomaviridae* family have been identified.

The latest demonstration of the oncogenic potential of a polyomavirus in humans, that has been ascribed to Merkel cell PyV (MCPyV), rekindled increasing interest in this viral family. MCPyV was isolated from the skin of a patient affected by Merkel Cell carcinoma (MCC) showing its ability to cause Merkel skin cancers [1]. However, the hypothesis that some among the hPyVs might play an etiological role in malignancies has been formulated more than 40 years ago [2]. Based on experimental models, the human polyomaviruses JC (JCPyV) and BK (BKPyV) have been recently categorized by the International Agency for Research in Cancer as “possible carcinogens”, although studies in humans showed inconsistent evidence for an association with cancers at various sites [3].

In this review, the hypothesis that JCPyV could play a role in the development of Central Nervous System (CNS) and colon tumors will be elucidated and in deeply analyzed, based on the results and the reports published in the most recent literature.

## JCPyV: epidemiology, structure, and life cycle

Humans are the natural hosts for JCPyV, that was isolated in 1971 from the brain tissue of a Hodgkin lymphoma patient, with initials J.C., who suffered from Progressive Multifocal Leukoencephalopathy (PML) [4].

JCPyV is ubiquitous and its primary infection, occurring during the childhood, is typically subclinical or linked to a mild respiratory illness. Between the age of 1 and 5 years, up to 50% of children show antibody to JCPyV, and by age of 10 years JCPyV seropositivity can be observed in about 60% of the population [5, 6]. By early adulthood, as many as 70–80% of the population has been infected [7]. Asymptomatic viral shedding in urine has been seen in both healthy and immunocompromised patients [8]. The mode of transmission for JCPyV is not yet well defined, although the presence of JCPyV DNA in B-cells and stromal cells of the tonsils and oropharynx supports the hypothesis of a respiratory route of transmission, with secondary lymphoid tissues serving as the potential site for initial infection [9]. Nevertheless, JCPyV was found also in raw sewage and in a high percentage of normal tissue samples taken from the upper and lower human gastrointestinal tract, suggesting that ingestion of contaminated water or food could be another portal of virus entry [10–13]. Moreover, JCPyV footprints have been reported in other many tissues of asymptomatic individuals, including spleen, lymph node, lung, bone marrow, brain, B lymphocytes and kidney, the last thought as the major site of JCPyV persistence.

The primary infection is followed by a lifelong, subclinical persistence of episomal viral genome in the cells. In the context of profound immunosuppression, the virus can become reactivated, leading to the lytic destruction of the oligodendrocytes, and the consequent development of PML, a fatal demyelinating disease [10]. It is not well assessed whether the immunosuppression of the host promotes the viral spread from the latency sites to the CNS or if JCPyV is already latent in the CNS and reactivates [11, 12].

The structure of the JCPyV virion is characterized by a non-enveloped, icosahedral capsid, measuring 40–45 nm in diameter and comprising 88% proteins and 12% DNA. The capsid is composed of three virus-encoded structural proteins, Viral Protein 1, 2, and 3 (VP1, VP2 and VP3). VP1 is the major component, with 360 molecules per capsid, and VP2 and VP3 contribute with 30–60 molecules each to the capsid. The icosahedron consists of 72 pentamers with no apparent hexamers, each composed of five VP1 molecules and one molecule of VP2 or VP3. Only VP1 is exposed on the surface of the capsid, and this determines the receptor specificity [13, 14].

The capsid surrounds a single, super-coiled, circular, double-stranded DNA molecule of 5130 base pairs (bp), in the case of the prototype JCPyV genome Mad-1 strain. The viral genome is associated with cellular histones H2A, H2B, H3 and H4 to form the so-called minichromosome, structurally indistinguishable from host cell chromatin; the viral particles do not contain linker histones, but the genome acquires them after entry into the host cell [13–15].

The viral genome of JCPyV is functionally divided into three regions, called the genetically conserved early and late coding regions, of about the same size, which are separated by the hypervariable non-coding control region (NCCR), containing the origin of viral DNA replication (ori), the TATA box, binding sites for cellular transcription factors and bidirectional promoters and enhancers for the transcription of early and late genes. The NCCR of JCPyV is the most variable portion of the viral genome within a single virus. Viral DNA transcription and replication occur bidirectionally starting from the NCCR: the early transcription proceeds in a counterclockwise direction, while the late transcription proceeds clockwise on the opposite strand of DNA [16].

The early coding region spans about 2.4 kb and encodes the alternatively spliced transforming proteins large tumor antigen (T-Ag) and small tumor antigen (t-Ag), which are involved in viral replication, and in promoting transformation of cells in culture and oncogenesis in vivo. Three additional proteins, named T’_135_, T’_136_ and T’_165_, due to the alternative splicing process are also produced at high level in the lytic cycle [17, 18]_._

T-Ag, a nuclear phosphoprotein of approximately 700 amino acids (aa), is considered the master regulator of the infectious process, because it orchestrates the production of early precursor messenger RNA (pre-mRNA), the initiation of viral DNA replication and the activation of late genes transcription. Moreover, by binding to the hypophosphorylated form of the pRb, T-Ag allows for premature release of the transcription factor E2F, which stimulates resting cells to enter the S-phase of the cell cycle.

T-Ag directly recruits the host cell DNA polymerase complex to the origin in order to initiate bi-directional DNA synthesis. Activation of the late viral promoter by T-Ag and associated cellular transcription factors lead to viral late gene expression [15].

t-Ag is a cysteine-rich protein of 172 aa, the first 80 of which are shared with T-Ag. t-Ag role in the lifecycle of JCPyV is not yet fully understood, though it is believed to serve an ancillary role for T-Ag activity and cell transformation [16, 19].

The late coding region spans 2.3 kb and contains the genetic information for the major structural protein VP1 and the two minor structural proteins VP2 and VP3, that are encoded from a common precursor mRNA by alternative splicing. The late region also encodes the Agnoprotein, a small multifunctional protein, that participates in viral transcriptional regulation, and inhibition of host DNA repair mechanism [20]. Additionally, JCPyV encodes a pre-microRNA (miRNA) that is processed into two unique miRNAs (JCPyV-specific miR-J1-5p and miR-J1-3p) during the late phase of infection. Both miRNAs are capable of downregulating the early phase protein T-Ag [21].

The infection of cell by JCPyV requires the binding between the viral VP1 and an N-linked glycoprotein with sialic acid: JCPyV uses both the α(2,3)- and α(2,6)-linked sialic acids to infect the permissive glial cells [22]. In addition, JCPyV is able to bind the serotonin receptor, 5HT2AR, that is present on cells in the brain and in the kidney, and to the ganglioside GT1b [23, 24]. Once the virus has gained entry into the host cell, by clathrin-dependent endocytosis [25], it travels to the cell nucleus, where it is uncoated and transcription of the early region begins. The early product T-Ag, back into the nucleus, binds to the viral origin of replication and allows the replication of the viral DNA, that depends by the availability of the cell DNA polymerase, replication protein A (RPA) and with host enzymes and cofactors, expressed in the S-phase of the cellular cycle [26]. As JCPyV replication proceeds, the late genes are expressed and the late products, VP1, VP2 and VP3 begin to assemble with the viral DNA, to form the complete virion. The final viral products are released via host cell lysis [27].

There is another possible outcome to infection of a cell by JCPyV: viral entry in nonpermisive cells, that do not support viral replication, can end up with the cell transformation or oncogenesis [28].

## Molecular mechanisms of JCPyV transformation mediated by T-Ag

The JCPyV principal actor, leading to cell transformation and tumor development, is the early protein T-Ag. T-Ag is a multifunctional protein, divided in several domains, defined, from the N-terminal to the C-terminal, as follows: the DNaJ domain, linking to the cellular factor HSc70; the LxCxE motif, that specifically binds and inactivates the Rb family members; the Origin-Binding Domain (OBD) that binds the JCPyV origin of replication; the NLS domain, that is necessary for the nuclear localization of the protein; the Helicase domain (containing the Zn and nucleotide binding domains), and, finally, the p53 binding domain [29, 30]. All these domains cooperate in binding to and inactivating cellular proteins that usually prevent the transition into S-phase; consequently, JCPyV itself, drives the cell cycle from G1 into S-phase. This event promotes viral replication and spread, when JCPyV infects permissive cells, while it drives to cell transformation, when JCPyV infects non permissive cells.

Basically, this progression is mainly the result of the binding between the T-Ag LxCxE motif (aa 103–107) and the members of the Rb tumor suppressor family [31–33]. T-Ag sequestration of the hypophosphorylated form of pRb enables the activation of the transcription factors E2F1, −2, −3a and 3b, that in turn activate the transcription of some genes, needed to enter the S-phase of the cellular cycle, such as *c-fos*, *c-Myc, cyclins A,D1* and *E, DNA polymerase alpha, thymidine kinas,* and others [29, 34–37]. The disruption of the complex pRb/E2Fs is mediated by the J domain of T-Ag, that binds to the Hsc70, a chaperone, increasing its ATPase activity when associated with T-Ag; the energy produced by the ATP hydrolysis is used to separate the pRb from the E2Fs [38, 39]. In addition, T-Ag can bind other members of the Rb family, that are p130 and p107 [40]. The p130-E2F4/5 association usually anchors a large repressive complex; T-Ag contributes to disrupt the complex p130-E2F4/5 and to release the brakes imposed on cell proliferation [41].

The C-terminal region of T-Ag contains the p53-binding domain [42]. P53 is a tumor suppressor, whose levels are usually kept very low. In conditions of stress, such as DNA damage or presence of oncogenes, p53 rapidly increases its transcription, the p53 protein is accumulated and the DNA repair mechanism or the cell apoptosis or senescence mechanisms are induced. When T-Ag binds and inactivates p53, the growth arrest and the premature cell death are avoided, while the cell cycle progression is favoured also in presence of DNA damage [43, 44].

Additionally, other cellular proteins, such as insulin receptor substrate 1 (IRS-1) [45], β-catenin [46, 47], the neurofibromatosis type 2 gene product [48] and the antiapoptotic protein survivin [49] are implicated in binding to JCPyV T-Ag.

IRS-1 is a membrane associated tyrosine kinase, which mediates both physiological and pathological responses in the cell. Activated IRS-1 triggers cell proliferation, and sends antiapoptotic signals. It has been shown that T-Ag is able to bind directly to the IRS-1 and to cause its translocation into the nucleus and that this event has important consequences in the homologous-recombination-directed DNA repair (HRR) mechanism. In normal conditions, the Insulin Growth Factor-I receptor (IGF-1R)/IRS-1 signaling axis supports HRR: the mechanism involves a direct binding between hypophosphorylated IRS-1 and Rad51 in the cytoplasm. Following IGF-IR stimulation, tyrosine phosphorylated IRS-1 loses the ability to complex Rad51, that translocates to the nucleus, where it participates in homology search and intrastrand invasion to support faithful DNA repair [50, 51]. Following T-Ag-mediated nuclear translocation, IRS-1 binds Rad51 at the site of damaged DNA and attenuates HRR. This indirect inhibition of HRR is associated with an increase number of cells accumulating mutations, that may be the base of the development of a malignant phenotype [45, 50, 52].

β-catenin is part of the Wnt pathway, that is involved in cell proliferation, survival and transcription processes. Several mutations in the proteins belonging to this pathway have been associated with the development of different tumors [53, 54]. T-Ag binds to β-catenin through the aa 82–628 and induces the stabilization of the cellular protein, whose levels increase [55]. Additionally, following the T-Ag interaction, β-catenin tranlocates into the nucleus and induces the transcription of *c-myc* and cyclin D1 [46].

The interaction between T-Ag and the neurofibromatosis type 2 (NF2) gene product and its translocation to the nucleus were also shown [48], but very few is known about the consequences of this association [56].

Finally, it has been observed that the binding between T-Ag and the antiapoptotic protein survivin leads to a significant decrement of the apoptotic process [49]. Reactivation of Survivin by JCPyV T-Ag can be a critical step in prolonging cell survival, which allows JCPyV to complete its replication cycle. Such a strong reactivation of the normally dormant Survivin has been observed in primary oligodendrocyte and astrocyte cultures infected in vitro, and expressing T-Ag. This can be a critical step in the transformation and proliferation of neural progenitors in vitro and in vivo [57].

**Fig. 1:**
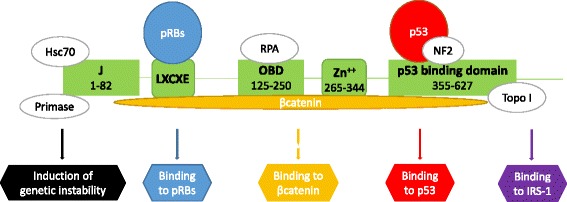
Molecular mechanisms of T-Ag induced- cell transformation. T-Ag binds to pRB family proteins, to βcatenin, p53 and IRS-1, inducing the expression of many genes involved in the advancement of the cell cycle and/or interfering with the apoptosis and the NHEJ double stranded DNA repair mechanism processes. Additionally, T-Ag promotes the induction of genetic instability

T-Ag has also a direct mutagenic effect on the host genome, by inducing spontaneous mutations in the infected cells and cytogenetic alterations, both influencing chromosomal stability and cell kariotype [58]. These damages may precede the morphological transformation [59] (Fig. 1).

The alternative T’ early proteins are also able to bind to the Rb family components, with a particular affinity with p107 (T’_135_ and T’_136_); moreover T’_135_ binds Hsc70 [31, 60].

## Molecular mechanisms of JCPyV transformation mediated by t-Ag

The t-Ag is encoded by the same mRNA that encodes the T-Ag, following a mechanism of alternative splicing. Consequently, the N-terminal 82 amino acids are the same as the N-terminus of T-Ag, while the C-terminus is an unique domain. The t-Ag is not studied as much as T-Ag and the majority of the information regarding its functions derives from what is known about the SV40 t-Ag. SV40 t-Ag cooperates with T-Ag to enhance transformation when T-Ag levels are low [61], it is required for human cells transformation [62], and is needed to keep high level of viral load in persistent infection of human mesothelial cells [63]. It has been demonstrated that, in contrast with SV40 t-Ag, JCPyV plays a relevant role in viral replication, since t-Ag null mutant failed to display detectable DNA replication activity [64].

**Fig. 2:**
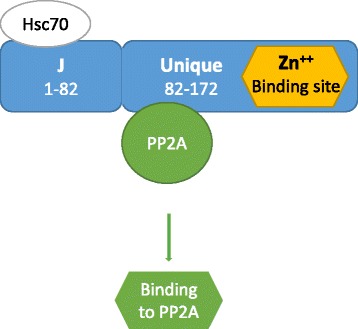
Molecular mechanisms of t-Ag induced- cell transformation. t-Ag binds to PP2A, activating several pathways that promote cell proliferation, including the MAPK pathway

The unique domain of the JCPyV t-Ag contains the binding site for the Protein Phosphatase 2A (PP2A), a serine/threonine –specific protein phosphatase that is involved in the mitogen-activated protein kinase (MAPK) pathway. The interplay between t-Ag and PP2A is also mediated by the JCPyV Agnoprotein and the result of this binding is an interference with the phosphatase activity of PP2A [65] and the subsequent activation of pathways inducing cell proliferation. Additionally, it has been shown that t-Ag binds to the members of the Rb family pRb, p107 and p130 and these associations are expected to influence cell cycle progression [64] (Fig. 2).

## Molecular mechanisms of JCPyV transformation mediated by Agnoprotein

**Fig. 3:**
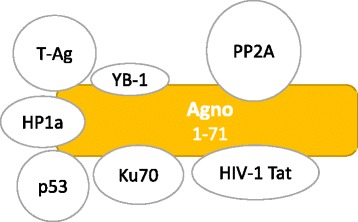
Molecular mechanisms of Agnoprotein induced- cell transformation. Agnoprotein binds to several viral and cell factors, such as T-Ag, HIV-Tat, p53, Ku70, PP2A, YB-1 dysregulating cell cycle progression

The JCPyV late genomic region encodes a regulatory protein, known as Agnoprotein. It is a very small protein of 71 aa in length, that was named “agno”, because when its encoding ORF was discovered, no protein was associated to it [66]. Agnoprotein is produced late in the infectious cycle, but is not incorporated into the mature virion; additionally, it is phosphorylated and it has been shown that the posphorylation is necessary for the functionality of the protein and the replication of the virus [67]. Over the years, JCPyV Agnoprotein was demonstrated to bind to both viral (T-Ag, t-Ag, VP1) and cellular (YB-1, p53, FEZ1, PP2A, Ku70…) proteins [65, 68–74]. Consequently, it plays a role in the viral transcription, translation, assembly and also in the cell cycle progression. In particular, Agnoprotein binds directly to p53 causing the arrest of the cell cycle in the G2/M phase due to the activation of p21/WAF-1 promoter [73]. The interaction of the Agnoprotein with Ku70 drives to the inhibition of the non homologous end joining (NHEJ) double stranded DNA repair mechanism, contributing to the genomic instability conferred on cells undergoing JCPyV infection [74]. As already explained before, Agnoprotein is phosphorylated, but the binding with PP2A causes its dephosphorylation; when PP2A is sequestered by t-Ag, it cannot act as a phosphatase on Agnoprotein, and this causes a downregulation of JCPyV replication, but also an activation of the MAPK signaling [65]. All together, the description of the characteristics of the Agnoprotein demonstrated its importance in the cellular transformation process [75] (Fig. 3).

## JCPyV oncogenicity in experimental animals

The highly oncogenic potential of JCPyV has been well established in different animal models, starting from 1973, when it has been shown that the inoculation of the virus into the brain of newborn Golden Syrian hamsters can lead to the development of unexpected tumors, such as medulloblastoma, astrocytoma, glioblastoma multiforme, primitive neuroectodermal tumors and peripheral neuroblastoma [2, 76, 77]. Astrocytoma, glioblastoma and neuroblastoma also developed after intracerebral inoculation of JCPyV into owl and squirrel monkeys [78]. Interestingly, the tumor tissues taken from the hamster and monkeys infected animals showed the presence of the T-Ag protein, but neither the expression of other virion antigens nor evidence of viral replication were found [79]. This is consistent with the fact that the animal cells may not be permissive for the JCPyV replication and leads to the consideration that JCPyV is able to transform the non permissive cells also in the human populations [80].

Other evidences regarding the JCPyV oncogenicity come from studies on transgenic mice, generated to contain the entire T-Ag coding sequence under the control of its own promoter, and without any other viral genes. Adrenal neuroblastoma, pituitary adenoma, malignant peripheral nerve sheat and medulloblastoma were the tumors induced by the expression of the only early protein [81–84].

## JCPyV and human CNS tumors

The ability of JCPyV to transform cells, such as human fetal glial cells and primary hamster brain cells, has been demonstrated in vitro. Furthermore, JCPyV was able to induce different types of brain tumors after injection in hamster, owl and squirrel monkeys [2, 85, 86]. Transgenic mice expressing the JCPyV early region were shown to develop adrenal neuroblastomas, tumors of primitive neuroectyodermal origin, tumors arising from the pituitary glan, glioblastoma multiforme, primitive neuroectodernal tumors and malignant peripheral nerve sheath tumors [28, 48, 80], and others.

All the molecular mechanisms previously described in this review appear to be involved in the JCPyV induced - neural oncogenesis, mainly due to the interaction of T-Ag with several cellular factors. Specifically, the binding between T-Ag and pRb promotes the cell cycle progression, while the T-Ag/p53 complex leads to the inhibition of the apoptosis process [28]; the interaction between the JCPyV early protein and IRS-1 or β − catenin is a key factor of the malignant transformation in children medulloblastoma [55, 87].

The first evidence of an association between the presence of JCPyV and a human tumor was reported in 1961, when Richardson [88], who first described PML, diagnosed an oligodendroglioma in a patient with concomitant chronic lymphocytic leukemia and PML. After the identification of JCPyV as the etiologic agent of PML, investigations focused on the possible association with brain tumors were conducted and at least ten cases were published, reporting the concomitant development of CNS neoplasia and PML [89, 90]. These clinical observations represent a strong proof that JCPyV may be involved in the pathogenesis of both the CNS diseases.

Detection of JCPyV sequences and/or protein expression in primary CNS malignancies has been frequently reported also in immunocompetent and/or immunosuppressed patients without PML. These reports regarded a wide variety of CNS neoplasia: gangliocytoma, choroid plexus papilloma, pilocytotic astrocytoma, subependymoma, pleomorphic xanthoastrocytoma, oligodendroglioma, all subtypes of astrocytoma, ependymoma, oligoastrocytoma, glioblastoma multiforme, medulloblastoma, pineoblastoma, gliosarcoma and primitive neuroectodernal tumors, as reported in Table 1.

**Table 1:** Detection of JCPyV in primary central nervous system tumor

Tumor	Reference	Detected/sampled (%)		Detection method	
		*DNA*	*Proteins*	*DNA*	*Proteins*
Adenocarcinoma	[143]	1/3 (33.3)	-	qPCR	-
Anaplastic Astrocytoma	[144]	6/15 (40.0)	-	qPCR	-
	[78, 145]	3/4 (75.0)	0/4 (0.0)	PCR, SB	IHC (T-Ag)
Anaplastic Ependynoma	[91]	0/1 (0.0)	-	PCR	-
Anaplastic Meningioma	[91]	0/1 (0.0)	-	PCR	-
Anaplastic Oligoastrocytoma	[144]	0/2 (0.0)	-	qPCR	-
Anaplastic Oligodendroglioma	[78, 145]	2/3 (66.7)	2/3 (66.7)	PCR, SB	IHC (T-Ag)
	[144]	3/8 (37.5)	-	qPCR	-
Astrocytoma	[146]	4/10 (40.0)	1/10 (10.0)	nPCR	IHC (T-Ag)
	[147]	1/3 (33.3)	1/3 (33.3)	nPCR, PCR	IHC (T-Ag)
	[78, 145]	10/16 (62.5)	7/16 (43.8)	PCR, SB	IHC (T-Ag)
	[148]	1/5 (20.0)	-	nPCR	-
	[144]	31/78 (39.7)	-	qPCR	-
	[144]	5/12 (41.7)	-	qPCR	-
	[143]	1/3 (33.3)	-	qPCR	-
	[149]	6/19 (31.6)	-	qPCR	-
	[150]	0/23 (0.0)	-	PCR	-
Chroid plexus papilloma	[151]	1/5 (20.0)	1/5 (20.0)	PCR, SB	IHC(T-Ag,Agno)
	[150]	0/14 (0.0)	-	PCR	-
Ependyomomas	[145]	5/6 (83.3)	4/6 (66.7)	PCR, SB	IHC (T-Ag)
	[151]	5/18 (27.8)	4/18 (22.2) 3/18 (16.7)	PCR, SB	IHC(T-Ag,Agno)
	[147]	0/2 (0.0)	0/2 (0.0)	nPCR, PCR	IHC (T-Ag)
	[146]	1/5 (20.0)	0/5 (0.0)	nPCR	IHC (T-Ag)
	[150]	1/34 (2.9)	-	PCR	-
	[148]	0/2 (0.0)	-	nPCR	-
	[143]	0/1 (0.0)	-	qPCR	-
	[149]	0/5 (0.0)	-	qPCR	-
Gangliocytoma	[147]	0/1 (0.0)	0/1 (0.0)	nPCR, PCR	IHC (T-Ag)
	[148]	0/1 (0.0)	-	nPCR	-
Gangliogioma	[149]	2/5 (40.0)	-	qPCR	-
Glioblastoma	[144]	20/51 (39.2)	-	qPCR	-
	[150]	2/102 (2.0)	-	PCR	-
	[148]	11/21 (52.4)	-	nPCR	-
	[149]	19/39 (48.7)	-	qPCR	-
Glioblastoma Multiforme	[78, 145]	12/21 (57.1)	5/21 (23.8)	PCR, SB	IHC (T-Ag)
	[152]	1/100 (1.0)	1/100 (1.0)	PCR, SB	IHC (T-Ag)
	[147]	7/13 (53.8)	7/13 (53.8)	nPCR, PCR	IHC (T-Ag)
	[153]	1/100 (1.0)	1/100 (1.0) 1/100 (1.0)	PCR	IHC(T-Ag,Agno)
	[143]	0/7 (0.0)	-	qPCR	-
Glioblastosis celebri	[78, 145]	1/100 (1.0)	1/100 (1.0)	PCR, SB	IHC (T-Ag)
Gliosarcoma	[149]	2/5 (40.0)	-	qPCR	-
Lymphoma	[149]	1/7 (14.3)	-	qPCR	-
Medulloblastoma	[154]	11/16 (68.8)	9/16 (56.3) 11/16(68.8)	PCR, SB	IHC (T-Ag)
	[155]	0/8 (0.0)	0/8 (0.0)	PCR, SB	IHC (T-Ag)
	[156]	11/23 (47.8)	4/23 (17.4)	PCR, SB	IHC (T-Ag)
	[157]	0/15 (0.0)	0/15 (0.0)	PCR, SB	IHC (T-Ag)
	[158]	-	0/22	-	IHC (T-Ag,Agno)
	[151]	0/32 (0.0)	0/32 (0.0)	PCR, SB	IHC (T-Ag)
	[143]	0/1 (0.0)	-	qPCR	-
	[149]	2/5 (40.0)	-	qPCR	-
	[150]	0/21 (0.0)	-	PCR	-
	[91]	0/2 (0.0)	-	PCR	-
Meningioma	[150]	0/15 (0.0)	-	PCR	-
	[148]	3/8 (37.5)	-	nPCR	-
	[91]	1/1 (100.0)	-	PCR	-
	[143]	6/12 (50.0)	-	qPCR	-
Oligoastrocytoma	[78, 145]	5/8 (62.5)	2/8 (25.0)	PCR, SB	IHC (T-Ag)
	[159]	1/100 (1.0)	1/100 (1.0)	PCR	IPPt (T-Ag)
	[143]	0/1 (0.0)	-	qPCR	-
	[149]	2/3 (66.7)	-	qPCR	-
	[144]	2/6 (33.3)	-	qPCR	-
Oligodendroglioma	[148]	1/2 (50.0)	-	nPCR	-
	[149]	4/12 (33.3)	-	qPCR	-
	[143]	0/2 (0.0)	-	qPCR	-
	[78, 145]	4/7 (57.1)	-	PCR, SB	-
	[160]	13/15 (86.7)	8/18 (44.4) 10/18(55.6)	PCR, SB	IHC (T-Ag,Agno)
	[147]	1/2 (50.0)	1/2 (50.0)	nPCR, PCR	IHC (T-Ag)
	[146]	1/5 (20.0)	0/5 (0.0)	nPCR	IHC (T-Ag)
	[144]	5/17 (29.4)	-	qPCR	-
Pilocytic Astrocytoma	[78, 145]	4/5 (80.0)	1/5 (20.0)	PCR, SB	IHC (T-Ag)
	[151]	0/7 (0.0)	0/7 (0.0)	PCR, SB	IHC (T-Ag,Agno)
Pineocytoma	[147]	0/1 (0.0)	0/1 (0.0)	nPCR, PCR	IHC (T-Ag)
	[143]	0/2 (0.0)	-	qPCR	-
	[149].	1/3 (33.3)	-	qPCR	-
	[148]	0/1 (0.0)	-	nPCR	-
Pituitary adenoma	[143]	0/3 (0.0)	-	qPCR	-
Pleomorphic xanthoastrocytoma	[161]	1/1 (100.0)	-	nPCR	-
Rare brain tumors	[149]	0/6 (0.0)	-	qPCR	-
Schwannoma	[143]	5/14 (35.7)	-	qPCR	-
sPNET	[157]	0/5 (0.0)	0/5 (0.0)	PCR, SB	IHC (T-Ag)
Subependymoma	[91]	0/1 (0.0)	-	PCR	-
	[78, 145]	1/1 (100.0)	1/1 (100.0)	PCR, SB	IHC (T-Ag)
Xanthoatrocytoma	[143]	0/1 (0.0)	-	qPCR	-

Legend: *qPCR* quantitative PCR, *nPCR* nested PCR, *IHC* immunohistochemistry, *SB* Southern Blot, *IPPt* immunoprecipitation, *sPNET* supratentorial primary neuroectodermal tumor

The percentage of JCPyV positive CNS tumor tissues was highly variable, ranging from 20 to 75%, with regard to the JCPyV genome and from 20 to 68% with regard to the JCPyV protein expression. Interestingly, the studies focusing on the viral protein expression were able to detect the viral early proteins T-Ag in the nuclei and Agnoprotein in the perinuclear area of the cells, but never the late VP1 protein (Table 1). These data are consistent with the fact that most of the CNS cells are non permissive for the JCPyV replication, and that the transforming ability of T-Ag appears limited to neural origin tissue.

Despite the increasing evidence of an association between JCPyV and the CNS tumors, it cannot be omitted that there is a lack of consistency in different studies that failed to detect both viral genome and protein expression in several types of tumors, such as meningioma [91], oligodendroglioma, astrocytoma [92], glioblastoma multiforme [93], glioma, and medulloblatoma [94]. Del Valle and colleagues hypothesized that the wide discrepancy in the viral genome and proteins detection, even within similar tumors, should be ascribed to the different types of collected samples, and to the employment of different techniques. They pointed out the fact that DNA isolated from formalin-fixed paraffin-embedded is usually of inferior quality than those isolated from fresh/frozen tissues and this may cause false negative results. The sensitivity of the routinary used amplification methods (PCR, nested PCR, quantitative-PCR, southern blot hybridization) is another important issue, that should be taken into account, since it can increase the rate of the false negative results [80].

The wide ubiquity of JCPyV, however, was demonstrated by the fact that some studies have underlined the presence of viral genomic sequences, but not DNA expression, also in brain from healthy immunocompetent subjects, with neither PML nor CNS malignancies [95–99].

This notable observation raises the question of whether the JCPyV found in CNS tumors may have a role in the pathogenesis of the malignancies or whether the brain is a latency site for JCPyV.

The model proposed by Perez-Liz [98] and colleagues and Del Valle and colleagues [80] made an effort in organizing all the puzzle pieces: following the primary infection, JCPyV establishes latency also in the brain and it does not replicate its genome neither express its proteins. In case of profound immunodepression, the virus can infect permissive cells, such as oligodendrocytes and induce a lytic cycle, exiting in the destruction of the infected cells and the subsequent development of PML. On the other hand, transient physiological changes may occur in normal individuals, allowing the expression of the T-Ag, and resulting in the accumulation of this oncogenic protein in brain cells. The result would be the interaction of T-Ag with the host proteins deputized to the cell cycle control, the promotion of uncontrolled cell division and the stimulation of tumor formation [100].

## JCPyV and human colorectal cancer

It is well assessed that JCPyV is commonly excreted in the urine of both immunocompetent and immunodepressed subjects and this is also demonstrated by the findings of JCPyV genome and complete virion in the raw urban sewage from around the world [101, 102] The ingestion of food and/or water contaminated with this virus easily leads to the infection of the gastrointestinal tract by JCPyV, whose structure is particularly resistant at very low pH (up to 1) in raw water [103, 104]. As described here below, an increasing number of studies, conducted worldwide, have reported the presence of JCPyV genomic sequences and the expression of T-Ag in tissues from gastrointestinal tumors, including esophageal carcinoma [105], gastric carcinoma [106–108], sporadic adenomatous polyps [109], and colorectal adenocarcinomas [110–117], but also in normal tissues and in adjacent noncancerous tissue from the gastrointestinal tract [118].

In the context of colorectal cancer, JCPyV seems to be a cofactor for the induction of the chromosomal instability [58, 119, 120], but it also interacts with the β-catenin protein with the consequent enhanced activation of Wnt pathway target genes, such as *c-Myc* and *Cyclin D1*. Both *c-Myc* and *Cyclin D1* are involved in cell cycle control and progression and their enhanced activation, mainly due to the intervention of T-Ag, could result in unchecked cell cycle progression, high proliferation rate, and ultimately a more malignant phenotype [46, 47, 121].

Overall, 18 different studies evaluated the presence of JCPyV in colorectal cancer, including studies that were aimed to identify only the viral genomic sequences or both viral genomic sequences and viral protein expression.

The first paper was published in 1999 by Laghi and colleagues and reported the presence of the T-Ag genomic sequence in 12 tissues samples out of 46 analyzed tissues (23 pairs of normal colorectal epithelium and adjacent cancers). The authors also showed that larger number of viral copies was present in cancer cells than in non-neoplastic colon cells [110]. The same research group also demonstrated some years later that 81.2% of normal colonic tissues and 70.6% of normal tissues from the upper gastrointestinal tract contained the T-Ag DNA sequences [104]. The presence of the JCPyV genome was confirmed by Enam and colleagues, who found 22 out of 27 tissues of malignant tumors of the large intestine positive for the presence of the T-Ag DNA; the expression of the oncogenic proteins T-Ag and Agnoprotein was observed only in 14 of these samples [46]. In adenomatous polyps of the colon, that are premalignant lesions, JCPyV T-Ag DNA sequences were found to be frequently present (82%), and T-Ag was found to be expressed specifically in the nuclei of 16% of these samples [109].

The remaining 14 studies evaluated the presence of JCPyV in colorectal cancer cases and controls. Eleven of them were extensively reviewed by Chen and colleagues in 2015 [118]. Additionally, a new case–control study was published in 2015, regarding JCPyV DNA in immunocompetent colorectal patients from Tunisia [117]. The remaining two studies focused on immunosuppressed patients and will be analyzed later [122, 123].

Taken together, ten papers reported the data obtained by the employment of Polymerase Chain Reaction (PCR), nested-PCR or quantitative PCR for the search of viral genomic sequences in a total of 746 colorectal cancer tissues and of 828 normal tissues (both adjacent noncancerous or tissues from healthy controls). Overall, 256/746 (34.3%) colorectal cancer tissues and 120/828(14.5%) were positive for the presence of the JCPyV genome [112, 115, 124–129]. Additionally 240 adenoma tissues were analyzed and compared with 257 normal tissues from healthy controls: JCPyV DNA was found in 77 adenoma (32.1%) and 48 normal (18.7%) tissues, respectively (Table 2) [115, 127, 128]. The expression of the JCPyV proteins was analyzed only in 4 studies [126, 130–132] and it has been observed that the early T-Ag protein was present in 9 out of 172 (5.2%) colorectal cancer or adenoma tissues and in 7 out of 38 (18.4%) adjacent noncancerous tissues or normal tissues from healthy controls (Table 3). Rollison and colleagues and Lundstig and colleagues collected blood samples from colorectal patients, and healthy controls and found a total of 210 (41.3%), and 179 (38.4%) seropositive subjects out of 509 colorectal patients, and 466 and healthy subjects (Table 3) [130, 131].

**Table 2:** Studies comparing JCPyV DNA prevalence between cases and controls

Reference	Positive cases/total cases (%) *Type of Sample*	Positive controls/total controls (%) *Type of Sample*
[125]	0/233 (0%) *CRC tumor tissue*	1/233 (0.4%) *Adjacent noncancerous tissue*
[128]	49/80 (61.3%) *CRC tumor tissue*	6/20 (30.0%) *Healthy tissue*
	15/25 (60.0%) *Adenoma tissue*	
[115]	6/23 (26.1%) *CRC tumor tissue*	0/20 (0%) *Healthy tissue*
	1/21 (4.8%) *Adenoma tissue*	
[126]	15/18 (8.3%) *CRC tumor tissue*	13/16 (81.2%) *Adjacent noncancerous tissue*
[112]	19/22 (86.4%) *CRC tumor tissue*	0/22 (0.0%) *Adjacent noncancerous tissue*
[129]	0/94 (0.0%) *Adenoma tissue*	0/91 (0.0%) *Healthy tissue*
[124]	56/137 (40.9%) *CRC tumor tissue*	34/137 (24.8%) *Adjacent noncancerous tissue*
		11/80 (13.8%) *Healthy tissue*
[127]	12/14 (85.7%) *CRC tumor tissue*	40/100 (40.0%) *Healthy tissue*
	55/60 (91.7%) *Adenoma tissue*	
[132]	38/114 (33.3%) *CRC glandular/stromal tissue*	2/20 (10%) *Healthy glandular/stromal tissue*
	6/40 (15.0%) *Adenoma glandular/stromal tissue*	
[117]	61/105 (58.1%) *CRC tumor tissue*	13/89 (14.6%) *Adjacent noncancerous tissue*

**Table 3:** Studies comparing JCPyV protein prevalence between cases and controls

Reference	Positive cases/total cases (%) *Type of Sample*	Positive controls/total controls (%) *Type of Sample*
[126]	9/18 (50.0%) *CRC tumor tissue*	7/18 (38.9%) *Adjacent noncancerous tissue*
[132]	0/114 (0.0%) *CRC glandular/stromal tissue*	0/20 (0.0%) *Healthy glandular/stromal tissue*
	0/40 (0.0%) *Adenoma glandular/stromal tissue*	
[131]	152/386 (39.4%) *CRC patient’s blood*	168/386 (43.5%) *Healthy subject’s blood*
[130]	58/123 (47.2%) *CRC patient’s blood*	11/80 (13.8%) *Healthy subject’s blood*

Interestingly, Selgrad and colleagues [122] and Boltin and colleagues [133] highlighted the important issue of JCPyV infection in the gastrointestinal tract in immunosuppressed patients. In particular, Selgrad and colleagues focused their attention on liver transplant patients who developed colorectal neoplasia and they showed that both the viral genome and early protein were present in higher percentage in colorectal mucosa and adenoma tissues from transplant patients than in non transplant patients. The hypothesis that has been formulated based on this finding was that the use of immunosuppressive agents may contribute in the reactivation of the virus and that the expression of T-Ag may represent a risk for the developing of neoplasia in immunosuppression conditions [122]. Similarly, Boltin and colleagues reported that JCPyV T-Ag DNA was more prevalent in the upper and lower gastrointestinal mucosa of 38 immunosuppressed patients than in the gastrointestinal mucosa of 48 immunocompetent subjects, possibly indicating that the virus resides in these patients. This may account for the higher prevalence of gastrointestinal carcinomas in immunosuppressed patients.

A very innovative starting point for the next research studies on the association between JCPyV and colorectal cancer comes from a recent publication, reporting that JCPyV specific miR-J1-5p miRNA could be used as a potential biomarker for viral infection in colorectal patients, since JCPyV miRNA lower expression was showed in the stools from patients with colorectal cancer, compared to healthy subjects [134]. However, the role of JCPyV miRNA in the development of the neoplasia remains to be elucidated.

Taken together, these reports demonstrated the presence of both JCPyV genome and proteins in tumor tissues, but also in the normal adjacent part or in normal colorectal mucosa and only in two studies the JCPyV prevalence was significantly higher in patients than in controls [112, 124]. Consequently, it is not possible yet to affirm whether JCPyV should be considered as an etiological cofactor, a risk factor or a simple bystander in the development of colorectal cancer. To this regard, Coelho and colleagues hypothesized that JCPyV might participate in different steps of the colorectal carcinogenesis: its latency might favor a transient inflammatory reaction, generating a microenvironment rich in cytokines, which can promote the expansion of transformed cells; the binding between T-Ag, Agnoprotein and several cell proteins might induce genetic instability, that can drive to irreversible genetic damages. The mechanism employed by JCPyV for inducing tumorigenesis might be the “hit and run”, where PyV infection is associated with the early stages of tumorigenesis, but is not needed for the progression of the disease, and this could explain why JCPyV genome/proteins were not always detected in the tumor tissues [135].

## Conclusions

Almost one fifth of human cancers worldwide are associated with infectious agents, either bacteria or viruses, and this makes the potential association between infections and tumors a relevant research issue. It is well assessed that the exposure to some viruses, such as Human Papillomavirus [136], Hepatitis B Virus [137], Human T leukemia virus [138] and MCPyV [1], can trigger the development of cervical carcinoma, liver carcinoma, leukemia and MCC, respectively. In this article, we have reviewed data concerning the possible link between JCPyV with CNS tumors and colorectal cancer.

Some of the biological features of JCPyV makes it a fully compatible candidate as risk factor of human tumors, because (a) it is usually acquired early in life; (b) it establishes a persistent infection in the host; (c) it encodes oncoproteins that interfere with tumor suppressors pathways, thus altering the normal progression of cell cycle; (d) it causes cancer in laboratory animals, and (e) viral sequences are often detected in human tumors. However, some other characteristics are not consistent with the known pattern of viral oncogenesis: it is ubiquitous in the human population and its genome/proteins can be easily detected in biological samples from healthy individuals; the length of infection is not determinable, since the primary infection is asymptomatic. In addition, it is well known that environmental and/or host cofactors could modulate the tumor pathogenesis, where viral infections could play a trigger role in the first step of transformation mechanism.

Some guidelines have been provided in order to prove cancer causation by a viral infection. JCPyV should have all the following requirements for being definitely associated to the development of CNS tumors and colon cancer: (a) the presence of its genome/proteins should be higher in cases than in controls; (b) the infection should always precede the disease symptoms; (c) the virus should have a highest prevalence in the geographical area where there is a highest prevalence of the tumor; (d) the virus should be able to transform human cell in vitro and to induce cancer in animal models [139, 140]. While JCPyV fulfills the second and the last criteria, it is difficult to apply the other two criteria to JCPyV: in fact it is ubiquitous in nature, but only a limited fraction of infected subjects develops disease; in addition, a variable time occurs between infection and the development of a cancer, making markers of exposure difficult to evaluate along the carcinogenic process [141]. Moreover, these criteria do not consider that some viruses, such as, probably, JCPyV may employ an “hit and run” oncogenic mechanism, where the virus induces cell transformation and, subsequently, is silenced or even lost during tumor progression [142].

At the light of all these observations, a causative role of JCPyV in human cancers is still to be defined, but, despite the “inadequate evidence of carcinogenicity in humans”, the WHO International Agency for Cancer Research Monograph Working Group decided to classify JCPyV as “possibly carcinogenic to humans”, belonging to group 2B, on the basis of the “sufficient evidence in experimental animals” [3]. Since the presence of JCPyV has been demonstrated in multiple human tumor tissues, it is reasonable to hypothesize that it could play a role as relevant cofactor in human tumorigenesis.

Therefore, only further solid, clear-cut epidemiologic, histopathologic and DNA evidence will ultimately settle this urgent issue and will help to answer the still unsolved question: “Does JCPyV cause tumors in the human population?” When a complete understanding is reached, a vaccination approach for the prevention of JCPyV infection may be proposed, based to the fact that JCPyV infection is acquired early in life and that, besides its possible transforming ability, this virus causes PML, a disease with no available and specific treatment.

## Abbreviations

aa: Amino acids
BKPyV: Human Polyomavirus BK
CNS: Central nervous system
hPyVs: Human polyomaviruses
HRR: Homologous-recombination-directed DNA repair
IGF-1R: Insulin Growth Factor-I receptor
IRS-1: Insulin receptor substrate 1
JCPyV: Human Polyomavirus JC
MAPK: Mitogen-activated protein kinase
MCC: Merkel cell carcinoma
MCPyV: Merkel cell PyV
miRNA: microRNA
mRNA: messenger RNA
NCCR: Non-coding control region
NF2: Neurofibromatosis type 2
NHEJ: Nonhomologous endjoining
OBD: Origin-Binding Domain
ori: Origin of replication
PCR: Polymerase chain reaction
PML: Progressive Multifocal Leukoencephalopathy
T-Ag: Large tumor antigen
t-Ag: Small tumor antigen
VP1, VP2, VP3: Viral Protein 1, 2, and 3
